# Mapping Transcriptome Data to Protein–Protein Interaction Networks of Inflammatory Bowel Diseases Reveals Disease-Specific Subnetworks

**DOI:** 10.3389/fgene.2021.688447

**Published:** 2021-08-18

**Authors:** Sefika Feyza Maden, Saliha Ece Acuner

**Affiliations:** Department of Bioengineering, Istanbul Medeniyet University, Istanbul, Turkey

**Keywords:** inflammatory bowel disease, ulcerative colitis, Crohn’s disease, protein–protein interaction networks, transcriptome

## Abstract

Inflammatory bowel disease (IBD) is the common name for chronic disorders associated with the inflammation of the gastrointestinal tract. IBD is triggered by environmental factors in genetically susceptible individuals and has a significant number of incidences worldwide. Crohn’s disease (CD) and ulcerative colitis (UC) are the two distinct types of IBD. While involvement in ulcerative colitis is limited to the colon, Crohn’s disease may involve the whole gastrointestinal tract. Although these two disorders differ in macroscopic inflammation patterns, they share various molecular pathogenesis, yet the diagnosis can remain unclear, and it is important to reveal their molecular signatures in the network level. Improved molecular understanding may reveal disease type-specific and even individual-specific targets. To this aim, we determine the subnetworks specific to UC and CD by mapping transcriptome data to protein–protein interaction (PPI) networks using two different approaches [KeyPathwayMiner (KPM) and stringApp] and perform the functional enrichment analysis of the resulting disease type-specific subnetworks. TP63 was identified as the hub gene in the UC-specific subnet and p63 tumor protein, being in the same family as p53 and p73, has been studied in literature for the risk associated with colorectal cancer and IBD. APP was identified as the hub gene in the CD-specific subnet, and it has an important role in the pathogenesis of Alzheimer’s disease (AD). This relation suggests that some similar genetic factors may be effective in both AD and CD. Last, in order to understand the biological meaning of these disease-specific subnets, they were functionally enriched. It is important to note that chemokines—special types of cytokines—and antibacterial response are important in UC-specific subnets, whereas cytokines and antimicrobial responses as well as cancer-related pathways are important in CD-specific subnets. Overall, these findings reveal the differences between IBD subtypes at the molecular level and can facilitate diagnosis for UC and CD as well as provide potential molecular targets that are specific to disease subtypes.

## Introduction

Inflammatory bowel disease (IBD) is a chronic disease with gastrointestinal tract inflammation, and there are approximately 6.8 million IBD cases worldwide (Jairath and Feagan, 2019; Meyers et al., 2020). Genetic factors have been scientifically proven to be effective in the onset of the disease, so individuals with a family history are likely to have the disease (Zhu et al., 2019). IBD can develop at all stages of life and last a lifetime, significantly reducing the quality of life of patients (Pugliese et al., 2020). Moreover, patients with IBD have a high risk of developing colon cancer (Pugliese et al., 2020). Although the cause of IBD is not known exactly, various environmental factors trigger the emergence and progression of the disease in genetically susceptible individuals. IBD includes two similar types of idiopathic bowel diseases, namely, ulcerative colitis (UC) and Crohn’s disease (CD), that differ in location and depth of involvement in the intestinal wall. The involvement in UC is limited to the large intestine, whereas CD may be involved in any part of the gastrointestinal tract from mouth to anus (Zhu et al., 2019; Winter and Weinstock, 2020). While these two subtypes share a variety of molecular pathogenesis, the macroscopic inflammation patterns are clinically different. Since UC and CD patients tend to show similar symptoms, appropriate diagnosis and treatment options may remain unclear. Improved molecular understanding can reveal disease-type-specific and even individual-specific targets (Vennou et al., 2019; Mitsialis et al., 2020); thus, it is important to reveal the molecular signatures of UC and CD at the network level.

In genomics studies, genes functioning in the epithelial barrier function and genes related to cellular innate immunity are found to be particularly associated with UC and CD, respectively (Fakhoury et al., 2014; Cohen et al., 2019). The NOD2 gene located at the IBD1 locus is the first gene associated with CD (Hugot et al., 2001). ATG16L1, which is necessary for autophagy in the cell, and STAT3 polymorphisms involved in cellular function by regulating gene activity are also associated with CD (Magalhaes et al., 2011; Wang et al., 2014). IRF5 polymorphisms (Gathungu et al., 2012) and ILR23 variants (Duerr et al., 2006) are associated with both UC and CD. Although most gene loci found for IBD show the same direction of action for UC and CD subtypes, some genes also have adverse effects. For example, while NOD2 and PTPN22 genes are risk factors for CD, they showed a significant protective effect for UC (Wawrzyniak and Scharl, 2018).

Understanding the complex mechanisms of diseases is important in both the diagnosis and treatment steps. In systems biology, the molecular understanding that develops with the analysis of biological networks can serve for disease type- and even individual-specific goals (Ran et al., 2013; He et al., 2017). One of the most effective approaches for disease-specific subnetwork (subnet) detection is the method of integrating transcriptome data into protein–protein interaction (PPI) networks (He et al., 2017). Transcriptome data include the whole gene transcripts (RNA molecules) expressed within the cell and represent the relationship between the information stored and encoded in DNA and the phenotype (Kaur, 2013). PPI networks, on the other hand, inform of the interactions between proteins in the organism (Rao et al., 2014). PPI networks and associated experimental data, different for each organism, can form a bridge between cellular processes and disease states (He et al., 2017). By mapping transcriptome data to PPI networks, proteins encoded by genes with significantly changed expression levels due to the relevant situation can be determined, and new modules can be obtained (He et al., 2017). This approach can reveal proteins and cellular mechanisms previously unknown to be related to the disease condition. Thus, with an integrated transcriptome and proteome analysis approach, the different mechanisms underlying network dynamics can be highlighted (Rakshit et al., 2014; Chen et al., 2016).

In a study comparing the performance of subnet discovery algorithms under different conditions, KeyPathwayMiner (KPM) (Alcaraz et al., 2014) has been reported to show high performance (Batra et al., 2017). KPM algorithm was also used to investigate the effect of chemotherapy (Warsow et al., 2013) and to understand the mechanism of Huntington’s disease (Alcaraz et al., 2011). Genes in the subnetwork modules, handled by KPM, are defined as significantly active genes in relation to the investigated situation (Warsow et al., 2013). In this paper, the expression data of IBD subtypes (UC and CD) were compared, and differentially expressed genes (DEGs) with respect to the healthy state were determined for each subtype. Moreover, disease subtype-specific PPI networks were extracted, topologically analyzed, and transcriptome data were mapped to PPI networks to identify UC- and CD-specific subnets. Biological significance of the disease-specific subnets was further explored by functional enrichment, revealing the differences between the IBD subtypes at the molecular level. The network-based pathway functional enrichment method can be used to discover molecular mechanisms related to diseases. With this approach, situation-specific functional modules are extracted from large interaction networks, and new modules are obtained and subjected to analysis (Batra et al., 2017). Our results can facilitate diagnosis for IBD subtypes UC and CD, and provide potential molecular targets.

## Materials and Methods

**FIGURE 1:**
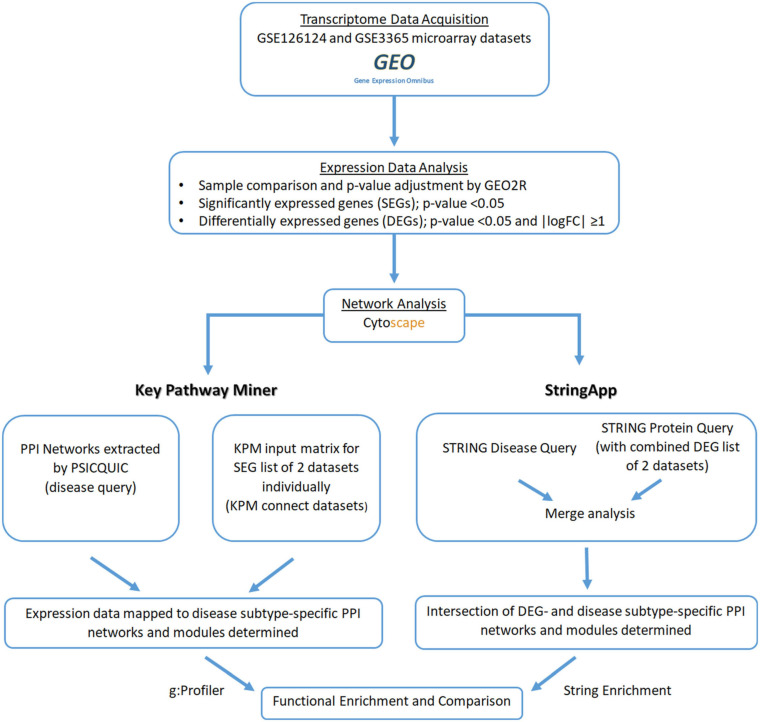
Workflow of the comprehensive protein–protein interaction (PPI) network analysis by mapping transcriptome data for the discovery of ulcerative colitis (UC)- and Crohn’s disease (CD)-specific subnetworks.

The expression data for IBD subtypes (UC and CD) were obtained from the GEO database, and differentially expressed genes (DEGs) with respect to the healthy state were determined for each subtype. All PPI network analyses and visualization were performed by the Cytoscape (3.8.0) application. Cytoscape (Shannon, 2003; Cline et al., 2007) is an open-source software for visualizing, modeling, and analyzing molecular and genetic interaction networks. Cytoscape can be applied to any molecular component and interaction system. In recent years, its use has increased with the emergence of large databases for protein–protein, protein–DNA, and genetic interactions of humans and model organisms (Shannon, 2003; Cline et al., 2007). In our study, two different approaches were used to map the transcriptome data to the disease subtype-specific PPI networks to identify UC- and CD-specific subnetworks (subnets) (Figure 1). The first approach uses KeyPathwayMiner (KPM) to determine the modules in the disease-specific PPI networks, whereas in the second approach, disease- and DEG-related PPI networks are merged using stringApp, and their intersection yields the disease-specific subnets. Last, biological significance of the disease-specific subnets, coming from different approaches, was explored by functional enrichment, revealing the differences between the IBD subtypes at the molecular level. The details of each step are explained in the following subsections, and the workflow of our comprehensive analysis of PPI networks with transcriptome data for the discovery of UC- and CD-specific subnetworks is shown in Figure 1.

### Acquisition and Analysis of Transcriptome Data

Transcriptome data for the IBD subtypes ulcerative colitis (UC) and Crohn’s disease (CD) were obtained from the Gene Expression Omnibus (GEO) database^1^ under microarray datasets GSE126124 and GSE3365 (Table 1). These microarray datasets were chosen because of the coexistence of samples from UC, CD, and normal (healthy) groups. The acquired transcriptome datasets were analyzed individually by comparing two disease conditions with the healthy condition such as UC vs. healthy tissue and CD vs. healthy tissue using GEO2R, a web tool that can perform R-based gene expression analysis. As default in GEO2R analyses, we applied quantitative normalization and the Benjamini and Hochberg procedure for controlling false discovery rate (FDR). This is the most frequently used adjustment for microarray data because of the good balance between limiting significant genes and false positives. Finally, for determining the differentially expressed genes (DEGs) that differ in mRNA level in UC vs. control and CD vs. control groups, the *p*-value threshold was considered as 0.05, and the direction of the change in gene expression was assigned according to fold change (FC) values. Genes with logarithm of fold change (logFC) value above 1 was considered as upregulated and below 1 as downregulated (|logFC| ≥ 1).

**TABLE 1:** Characteristics of the expression profiling microarray datasets GSE126124 and GSE3365 analyzed in this study.

Datasets	Ulcerative colitis (UC)	Crohn’s disease (CD)	Normal controls
GSE126124	36 samples	76 samples	51 samples
GSE3365	26 samples	59 samples	42 samples

### Identifying Ulcerative Colitis- and Crohn’s Disease-Specific Subnets

#### Mapping Transcriptome Data to Disease-Specific Protein–Protein Interaction Networks With Cytoscape—KeyPathwayMiner

Disease-specific PPI data for UC and CD were extracted using the PSICQUIC web service (October 29, 2020).^2^ Then the KeyPathwayMiner (KPM)^3^ (version 5.0.1) plugin for Cytoscape was downloaded. The KPM plugin of Cytoscape is able to efficiently uncover all the maximum connected subnets in a biological network. The KPM algorithm processes transcriptome data, *p*-values as “1” or “0” (Alcaraz et al., 2016), so *p*-values are arranged as “1” for significantly expressed genes (SEGs) (with *p*-value < 0.05) and “0” for non-significantly expressed genes, respectively. The *K*-value, which is an important parameter for KPM, indicates how many nonsense genes will be in the module. The optimal *K*-value was determined as 5 by trial and error (Alcaraz et al., 2016), such that significant genes are missed when the *K*-value is below 5, and no new significant genes are added when above 5. In this study, after loading the disease-specific PPI networks to Cytoscape, genes and *p*-values obtained from GSE126124 and GSE3365 datasets were arranged in the appropriate format for KPM and uploaded as two separate files. The *K*-value was assumed to be 5; the transcriptome data in the two sets were logically connected using “AND” and mapped to the PPI network so that new modules for UC and CD were obtained.

#### Merge Analysis of Differentially Expressed Gene- and Disease-Specific PPI Networks With Cytoscape—StringApp

The STRING database is designed to comprehensively combine, evaluate, and disseminate protein–protein relationship information (Franceschini et al., 2012). With StringApp^4^ (version 1.6.0), PPI queries can be performed in four different ways: protein query, PubMed query, disease query, and protein/compound query. In this study, the PPI network of DEGs in the two datasets was constructed using the STRING database, and an interaction with a composite score of > 0.95 was considered as statistically significant. Also, an interaction with a maximum protein count of 500 and a composite score of > 0.95 was considered statistically significant when querying the disease names (ulcerative colitis and Crohn’s disease) in the STRING database. In this way, DEG- and disease-specific PPI networks for UC and CD were merged (intersection analysis) with the Cytoscape application, and new intersection modules were obtained.

### Functional Enrichment of Disease-Specific Network Modules

#### Disease Subtype-Specific Modules Obtained by KeyPathwayMiner

Last, the new modules obtained with KPM were functionally analyzed using g:Profiler (Reimand et al., 2016),^5^ which is an online, user-friendly, and comprehensive database for functional enrichment analysis. It contains the methods commonly used in standard pipelines of biological entity (gene/protein)-centered computational analysis. g:Profiler currently includes Gene Ontology for biological pathway analysis; KEGG, Reactom, and WikiPathways, to determine the regulatory motifs in DNA; TRANSFAC and miRTarBase for protein databases; and it contains commonly used data sources such as the Human Protein Atlas and CORUM (Raudvere et al., 2019). Gene lists in the new disease subtype-specific modules obtained for UC and CD were given as input to g:Profiler algorithm; Benjamini–Hochberg was applied as the statistical correction method, and terms with *p*-values less than 0.05 were considered as significant.

#### Disease Subtype-Specific Modules Obtained by StringApp

String Enrichment can perform functional analysis of the modules created by STRING, which performs overrepresentation tests for a total of 11 functional path classification frameworks. Some commonly available frameworks are: Gene Ontology, KEGG paths, UniProt keywords, and Reactome paths (Szklarczyk et al., 2020). Functional enrichment by String Enrichment was applied on the intersection module obtained after the merge analysis of DEG- and disease-specific PPI networks for UC and CD, and the results were compared.

## Results and Discussion

### Functional Enrichment of Disease-Specific Differentially Expressed Genes

**FIGURE 2:**
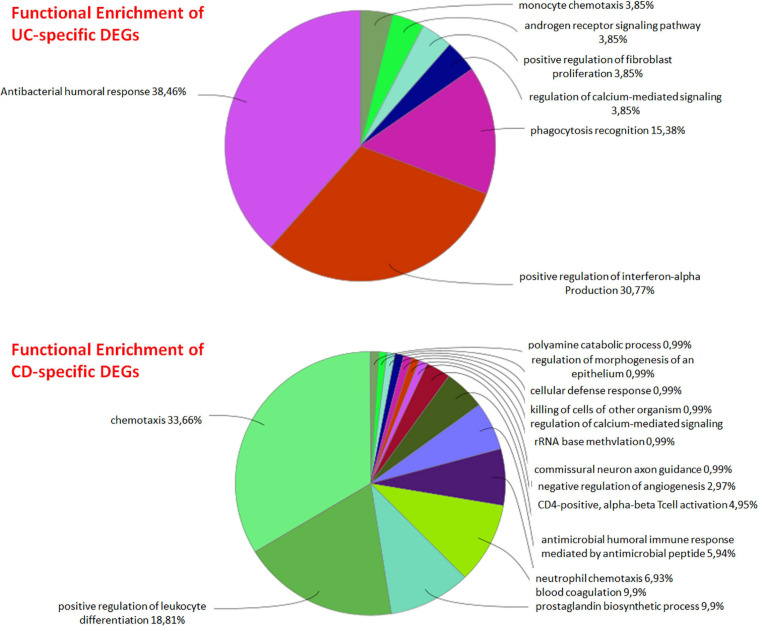
Functional enrichment of UC- and CD-specific differentially expressed genes (DEGs).

DEG analysis of GSE126124 and GSE3365 datasets resulted in 144 DEGs (84 upregulated and 60 downregulated) common for UC and CD, 136 UC-specific DEGs (68 upregulated and 68 downregulated), and 195 CD-specific DEGs (107 upregulated and 88 downregulated) (Tables 2, 3). Literature search of common up- and downregulated disease-specific genes confirms their importance in IBD. The regenerative gene (REG) family shows increased expression during IBD-associated inflammation (Xu et al., 2019). OLFM4 secreted by human intestinal epithelial cells is upregulated in the inflamed mucosa of IBD patients; however, its functional role in IBD has remained uncertain (Kuno et al., 2021). DMBT1 gene, which is considered as a candidate tumor suppressor gene for the brain, lungs, stomach, and colorectal cancers, has shown an increased expression in inflamed tissues of IBD patients, and it has been stated that impaired DMBT1 function may be associated with the onset of Crohn’s disease (Renner et al., 2007). Moreover, polymorphisms/mutations of Toll-like receptors (TLR), which are innate immune receptors, have been directly linked to IBD (Lu et al., 2018), and activation of epithelial TLR4 in IBD and colorectal cancer has been associated with upregulation of DUOX2 (Burgueño et al., 2020). It has been reported that MARK2, a master regulator of cell polarity in intestinal epithelial cells, may contribute to the initiation and progression of IBD by interfering with the protein kinase cascade (Yuan et al., 2017). Disease-specific DEGs were further analyzed by functional enrichment using ClueGO, as explained below (Figure 2).

**TABLE 2:** Top five upregulated differentially expressed genes (DEGs) and their corresponding *p*-values and logFC values of the ulcerative colitis-specific, Crohn’s disease-specific, and common genes.

UC-specific			CD-specific			Common genes		
Gene symbol	*p*-value	logFC	Gene symbol	*p*-value	logFC	Gene symbol	*p*-value	logFC
RNU5D-1	1.49E–03	1.9	FCGR1CP	3.16E–05	1.5	REG1B	1.24E–04	2.3
MMP12	1.87E–02	1.5	CXCL9	7.52E–04	1.3	REG1A	1.23E–03	2.2
PTCH2	1.55E–02	1.4	ANKRD22	4.99E–07	1.3	DUOX2	2.14E–05	1.9
TNFRSF17	6.13E–05	1.4	CD274	7.34E–10	1.3	OLFM4	4.76E–03	1.7
SAA1	1.26E–05	1.4	CXCL10	5.72E–03	1.2	DMBT1	2.79E–03	1.6

**TABLE 3:** Top five downregulated DEGs and their corresponding ***p***-values and logFC values of the UC-specific, CD-specific, and common genes.

UC-specific			CD-specific			Common genes		
Gene symbol	*p*-value	logFC	Gene symbol	*p*-value	logFC	Gene symbol	*p*-value	logFC
LIMD1-AS1	2.35E–05	−1.2	XCL2	2.12E–11	−1.8	NPRL2	1.12E–11	−2.2
KLF6	8.95E–09	−2.7	KLRF1	2.11E–12	−1.7	MYCBP2	8.41E–08	−2.1
DDX24	3.37E–06	−1.8	MYOM2	1.87E–05	−1.6	CLIC3	1.03E–08	−2.1
SART3	1.21E–05	−1.5	MIR664B	2.57E–14	−1.6	MARK2	5.09E–09	−1.9
KMT2A	2.94E–07	−1.5	HNRNPH1	1.13E–07	−1.6	SNRNP70	7.68E–13	−1.8

UC-specific genes were found to be mainly associated with signaling pathways such as antibacterial humoral response, positive regulation of interferon-alpha production, and phagocytosis recognition. Considering the antiviral effect of interferon alpha and the importance of phagocytosis in the early stage of bacterial infections, it can be said that UC-specific genes are effective on various immune system signaling pathways. Although the etiology of UC is not exactly known, it is assumed that it is a multifactorial condition that causes immune response (Zhang et al., 2017; Tatiya-Aphiradee et al., 2018). The immune response plays an important role in the initiation and progression of UC, and any loss of immune tolerance results in inflammation (Tatiya-Aphiradee et al., 2018). The relationship between CD-specific genes and chemokines and again the immune system was observed. It has been reported that the immunoregulatory effects of cytokines play an important role in the pathogenesis of IBDs such as Crohn’s disease (CD), where they control many aspects of the inflammatory response (Stallmach et al., 2004; Neurath, 2014).

### Identified Ulcerative Colitis- and Crohn’s Disease-Specific Subnets

#### Mapping Transcriptome Data to Protein–Protein Interaction Networks With Cytoscape—KeyPathwayMiner

**FIGURE 3:**
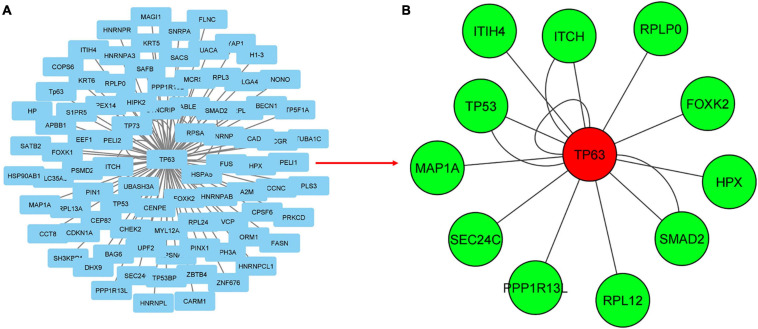
Mapping transcriptome data to UC-specific PPI network for the discovery of subnetworks with KeyPathwayMiner (KPM). **(A)** PPI network for UC obtained from the PSICQUIC database, including 88 proteins and 100 interactions. **(B)** Mapping transcriptome data to UC-specific PPI network reveals a module containing 13 proteins and 16 interactions. Green and red colored nodes correspond to the DEGs in the expression dataset and the genes that are not significantly different from the healthy samples, respectively, whereas circle-shaped nodes indicate they were mapped to both of the expression datasets.

**FIGURE 4:**
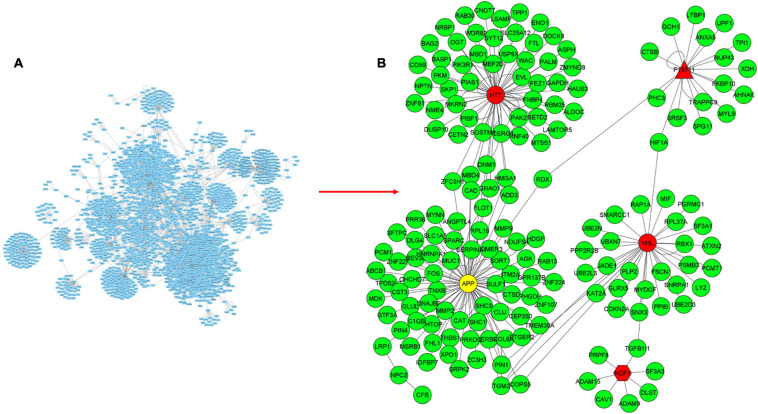
Mapping transcriptome data to CD-specific PPI network for the discovery of subnetworks with KPM. **(A)** PPI network for CD obtained from the PSICQUIC database, including 2777 proteins and 3475 interactions. **(B)** Mapping transcriptome data to CD-specific PPI network reveals a module containing 175 proteins and 217 interactions. Green and red colored nodes correspond to the DEGs in the expression datasets and the genes that are not significantly different from the healthy samples, respectively. Genes depicted in yellow are DEGs in only one of the datasets. Circle-shaped nodes were mapped to both of the expression datasets, nodes with pentagon shape were mapped to only one of the datasets, and triangle nodes were not mapped to any of the datasets.

The visualization and topological analysis of the disease-specific PPI network modules extracted from the PSICQUIC database were done by Cytoscape. UC-specific PPI module contains 88 proteins and 100 interactions (Figure 3A). Topological analysis revealed that there is only one hub gene (TP63) in the UC module. UC transcriptome data were mapped to the corresponding UC PPI network to find disease-specific new modules by KPM analysis (Figure 3B). The resulting UC-specific new module in the PPI network includes 11 nodes mapped to the DEGs (green circles) and one node (TP63, red circle) added by KPM was in the expression dataset but not found as a DEG (Figure 4B). The hub node TP63 (with the highest number of connections) is in the same family as the tumor proteins p53 (TP53) and p73 (TP73), which have been studied for risk associated with both colorectal cancer and inflammatory bowel disease (IBD) (Hudspath et al., 2018). Note that the existence of this hub node in the expression dataset but not being differentially expressed in disease conditions might be due to its vital role in various cellular processes and the network not being robust to changes in its expression levels.

CD-specific PPI module contains 2,777 proteins and 3,475 interactions (Figure 4A). In the new CD-specific module obtained by KPM analysis, 170 nodes were mapped to DEGs (green circles), and the APP hub node (yellow circle) was mapped to both transcriptome datasets but was significantly expressed in only one. Note that NOD2 gene, which is known to be related with CD, was not included in the modules, and this may be due to the fact that the NOD2 gene is not subtype-specific but rather generally associated with IBD. HTT and VHL hub nodes (red circle) were mapped to both transcriptome datasets but were not significantly expressed. The NCF1 hub node (red pentagon) was mapped to only one transcriptome dataset but was not significantly expressed. P19711 hub node (red triangle) could not be mapped to the transcriptome datasets (Figure 4B). Similar to the UC-specific subnet, hub genes were mapped to the expression dataset but not differentially expressed in the disease condition; indicating them to be critical for the network robustness. These CD-specific hub nodes and their disease relation can be listed as APP in Alzheimer’s disease (Neha et al., 2008), HTT in Huntington’s disease (HD) (Warby et al., 2011), and VHL as a tumor suppressor gene (Kim, 2004). Topological analysis reveals that the APP was the hub gene with the highest number of interactions with the expressed genes in the datasets. In addition, a recent study reported that APP gene is an important DEG for CD disease (Li et al., 2020). These results suggest that some similar genetic factors may be effective in both Alzheimer’s disease (AD), and CD as APP is a critical gene in both. Functional interpretation of the modules is given in the functional enrichment section.

#### Merged Differentially Expressed Gene- and Disease-Specific Protein–Protein Interaction Networks Using Cytoscape—StringApp

**FIGURE 5:**
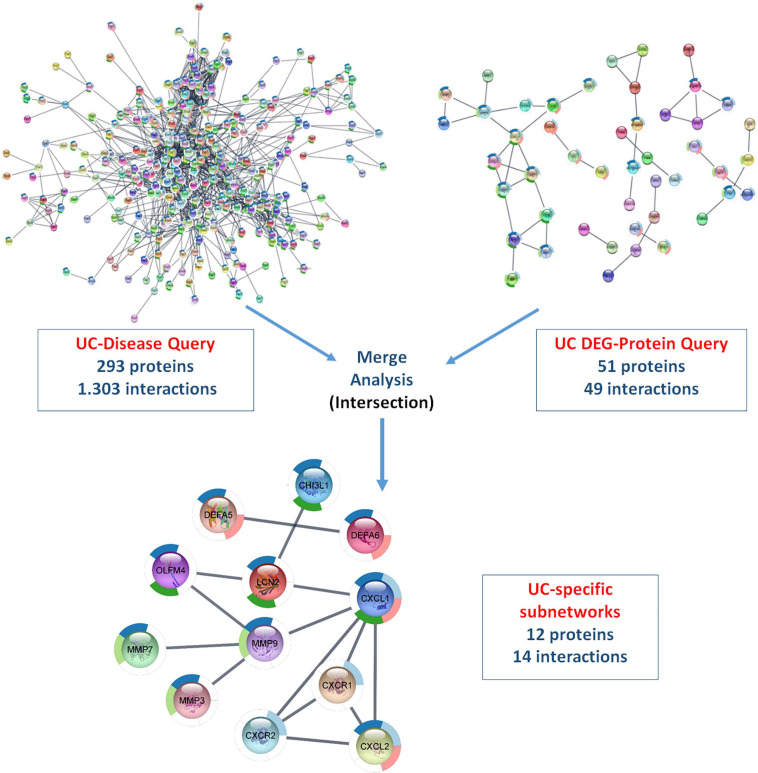
Analyzing disease- and DEG-specific PPI networks for the discovery of a UC-specific subnetwork with StringApp.

**FIGURE 6:**
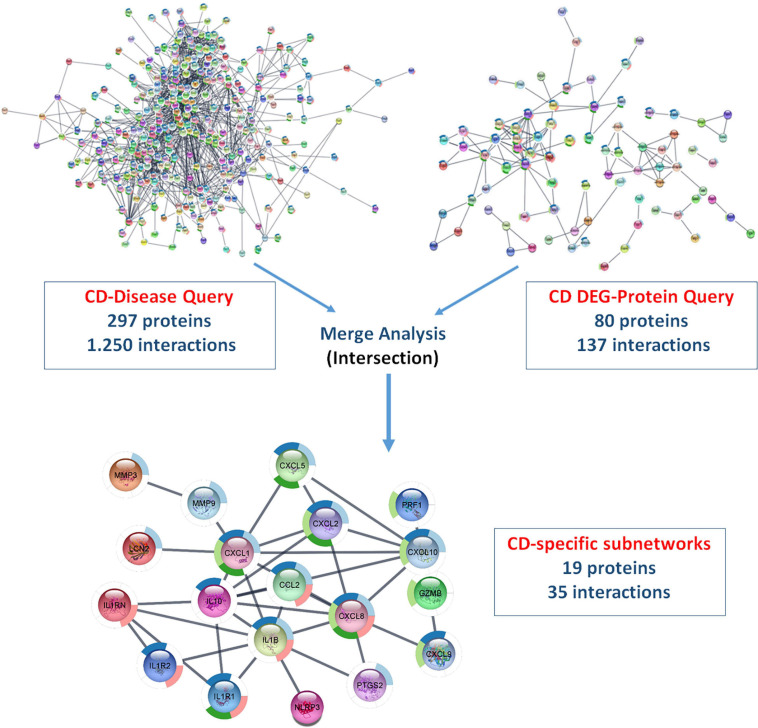
Analyzing disease- and DEG-specific PPI networks for the discovery of a CD-specific subnetwork with StringApp.

Using ulcerative colitis as the String Disease Query, a PPI network containing 500 proteins and 1,318 interactions was retrieved. When the non-interacting proteins with the rest of the network were removed, 293 proteins and 1,303 interactions remained in the UC-specific PPI network. In the String Protein Query search, a total of 280 UC-specific DEGs, including 152 up- and 128 downregulated genes, were used. As a result, a PPI network containing 246 proteins and 51 interactions was obtained, and removing the non-interacting proteins resulted in a DEG-specific PPI network of 51 proteins and 49 interactions. These two networks were subjected to merge analysis using Cytoscape, and the intersection of the two modules revealed a UC-specific subnet module containing 12 proteins and 14 interactions (Figure 5). A similar approach was also used for the discovery of a CD-specific subnetwork and the resulting number of proteins and interactions in the PPI networks are shown in Figure 6. Note that, Crohn’s disease as the String Disease Query, yielded 500 proteins and 1,263 interactions, which reduced to 297 proteins and 1,250 interactions after the elimination of the non-interacting nodes with the rest of the network. On the other hand, using a total of 339 CD-specific DEGs, including 191 up- and 148 downregulated genes, a PPI network containing 317 proteins and 144 interactions was obtained, and removal of non-interacting proteins with the rest of the network resulted in 80 proteins and 137 interactions. The CD-specific subnet module, which is the intersection of the disease- and DEG-specific PPI networks, includes 19 proteins and 35 interactions (Figure 6).

### Functional Enrichment of Disease-Specific Subnets

**FIGURE 7:**
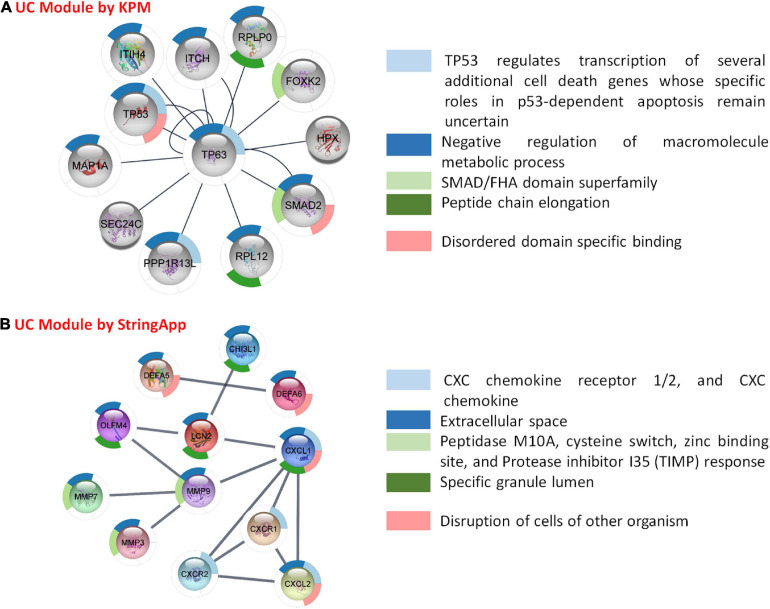
Functional analysis and comparison of UC-specific modules. **(A)** UC-specific module by KeyPathwayMiner (KPM) and the top 5 enrichment results based on the p-value. **(B)** UC-specific module by StringApp and the top 5 enrichment results based on the p-value.

**FIGURE 8:**
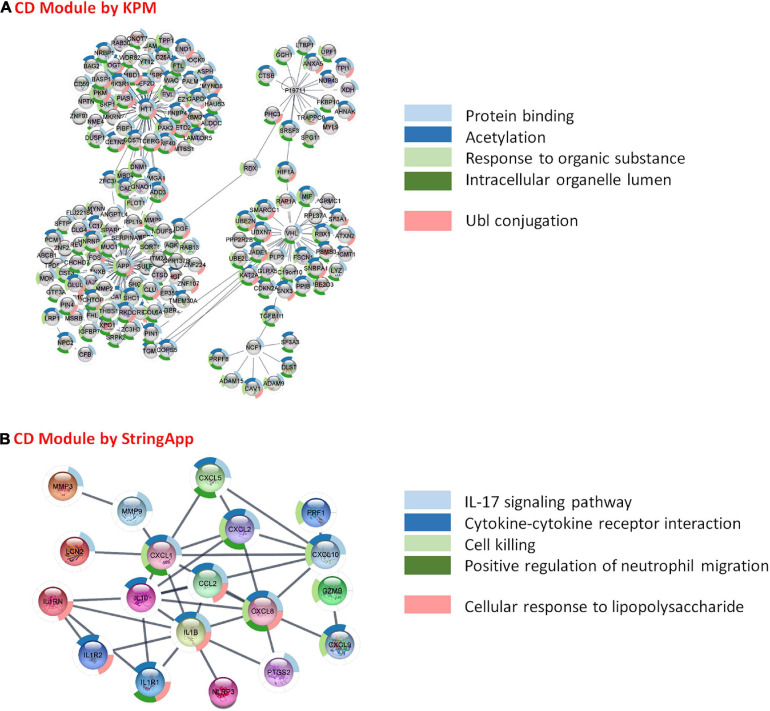
Functional analysis and comparison of CD-specific modules. **(A)** CD-specific module by KeyPathwayMiner (KPM) and the top 5 enrichment results based on the p-value. **(B)** CD-specific module by StringApp and the top 5 enrichment results based on the p-value.

As described above, the disease-specific subnets for UC and CD were obtained integrating transcriptome data into PPI networks using two different methods (Figures 1, 7, 8). In order to understand the biological meaning of these disease-specific subnets, they were functionally enriched. The functional enrichment of UC-specific module obtained by KPM showed that these genes mostly play a role in the regulation of the activity of cancer suppressor TP53 (Figure 7A). Studies have reported the presence of p53 overexpression in UC patients (Lu et al., 2017). Moreover, p53 expression is closely associated with colon cancer development in UC patients, and the prevalence of TP53 is reported to be high in patients with UC and colon cancer (Yashiro, 2014; Du et al., 2017; Lu et al., 2017). Most studies confirm that UC is a risk factor for colon cancer (Yashiro, 2014; Choi et al., 2016; Kobayashi et al., 2017). UC-associated colon cancer develops through the inflammation-dysplasia sequence, so early detection of any malignancy formation in patients with UC is very important (Kobayashi et al., 2017). The p53 protein overexpression as a result of the mutation of the p53 gene or the development of dysplasia can be used as a biomarker in the diagnosis of UC-associated colon cancer (Du et al., 2017; Kobayashi et al., 2017). On the other hand, the functional enrichment of the UC-specific module obtained by StringApp revealed that genes mostly play a role in the chemokine and disruption of cells of other organisms (Figure 7B). Chemokines are small cytokines secreted by cells that play a role in immunity and inflammation (Griffith et al., 2014). The UC-specific modules obtained by the two different approaches reveal common functional properties such as CXC chemokine receptor 1/2, and CXC chemokine, cellular response to transforming growth factor beta (TGF-beta) stimulus, T-cell proliferation involved in immune response, and identical protein binding. The following literature findings support these signature pathways in UC. CXC chemokine receptors 1/2 (CXCR1 and CXCR2) have similar signaling mechanisms (Muthas et al., 2016). Ligands of CXC chemokine receptor 1/2, which are chemoattractants of PMN (polymorphonuclear leukocyte), have been found at elevated levels in the mucosa of UC patients and the activation of PMN to the colonic mucosa causes tissue damage in patients with UC (Buanne et al., 2007). There is increasing evidence to suggest that CXCL8 (IL-8) has an important role in the pathogenesis of IBD (Williams et al., 2000). CXCL8 binds to CXCR1 and CXCR2 to mediate neutrophil recruitment and trigger cytotoxic action at the sites of infection (Nasser et al., 2009; Fisher et al., 2019). As a result of some studies, it was stated that the mucosal levels of CXCL8 were elevated in UC, but not observed in CD (Mahida et al., 1992; Bruno et al., 2015), and CXCL8 has been reported to mediate inflammation in UC (Zhu et al., 2021). Cytokine TGF-beta has critical functions for the fibrosis process such that it regulates the genes involved in wound healing, including enhancing extracellular matrix (ECM) formation, disordering the ECM cycle, and in the growth of connective tissue and insulin (Burke et al., 2007; Li and Kuemmerle, 2014; van Haaften et al., 2020). UC disease is characterized by cytokine production and T-cell infiltration (Koch Hansen et al., 2014). The pathogenesis of UC is associated with differences in immune regulatory T cells (Watanabe et al., 1997). Known for its anti-inflammatory roles, IL-10 cytokine has an important role in suppressing the exacerbation of disease symptoms of UC (Wang et al., 2020). Since IL-7 has an important role in the proliferation and differentiation of T cells, it contributes to the disruption of immune regulatory T cells in UC (Watanabe et al., 1997).

As a result of the functional enrichment of the CD-specific module obtained by KPM, general biological pathways have mainly emerged. These are, namely, protein binding, acetylation, response to organic substance, intracellular organelle lumen, and Ubl conjugation (Figure 8A). On the other hand, functional enrichment of CD-specific module obtained by StringApp results in functional pathways, such as IL-17 signaling pathway, cytokine–cytokine receptor interaction, cell killing, positive regulation of neutrophil migration, and cellular response to lipopolysaccharide (Figure 8B). The following literature findings support these signature pathways in CD. Interleukin-17 (IL-17) is determined to be the main immunoregulatory cytokine that can cause IBD with their disturbances (Hudspath et al., 2018). Due to its importance in the widespread expression of IL-17, the expression of various cytokines and chemokines are stated to be induced (Kim, 2004). The CD-specific modules obtained by the two different approaches reveal common functional properties such as cytokine production, interleukin-1 (IL-1) receptor activity, NF-kappa B (NF-κB) signaling pathway, bladder cancer, and growth factor activity. In CD patients, greater cytokine release and tissue damage were observed in inflamed tissues compared with non-inflammatory tissues (Sarrabayrouse et al., 2020). Interleukin-1 (IL-1) is one of the cytokines that promote inflammation (Dinarello, 2019). IL-1α and IL-1β are pro-inflammatory cytokines with similar structures; they bind to the same receptor and are present in different signaling pathways such as JNK, with NF-κB as the main active pathway (Dinarello, 2011; Garlanda et al., 2013; Anka Idrissi et al., 2021). Dysregulation of the NF-kB signaling pathways involved in regulating the immune response and inflammation is directly related to CD disease (Shih and Targan, 2007; Buttó et al., 2015; Han et al., 2017; Nissim-Eliraz et al., 2021). As a result of abnormal activation of NF-κB, overproduction of proinflammatory cytokines that cause chronic inflammation in the gut occurs (Han et al., 2017). Studies for CD patients have observed a significant increase in the number of NF-kB-positive cells in the inflamed area compared with the non-inflamed areas (Ellis et al., 1998). NF-κB activation status may reflect the inflammatory load in CD: CD patients with high NF-κB activation showed specific clinical signs such as higher frequency of ileocolonic involvement and lower frequency of perianal involvement compared with patients with low NF-κB activation (Han et al., 2017). Studies have shown that patients with CD are more likely to have bladder cancer than patients with UC (Geng and Geng, 2021; Zhang et al., 2021).

## Conclusion

Integrating transcriptome data into PPI networks to obtain disease-specific subnetworks (modules) approaches, which can be used to understand the complex natures of diseases, reveals previously unknown relations of proteins and cellular mechanisms with diseases. UC and CD are subtypes of IBD. The diagnosis and treatment processes of these diseases still remain unclear due to the complexity in the pathogenesis. In this study, we identified UC- and CD-specific subnets by mapping transcriptome data to PPI networks in order to reveal the molecular signatures and important functional pathways of these IBD subtypes. First, the analysis of GSE126124 and GSE3365 expression datasets showed UC- and CD-specific genes that significantly differ in mRNA level with respect to healthy cases (DEGs). Functional enrichment of these DEGs revealed that UC-specific genes act on various immune system signaling pathways, such as antibacterial humoral response, positive regulation of interferon-alpha production, and phagocytosis recognition. On the other hand, CD-specific genes were observed to be related with chemokines and again with the immune system. Then, new modules specific to CD and UC disease subtypes were identified employing two different approaches. As a result of the topological analysis of UC- and CD-specific modules obtained by KPM, TP63 was identified as the hub gene in the UC-specific subnet, and p63 tumor protein is studied for risk associated with both colorectal cancer and IBD being in the same family as p53 and p73. APP was identified as the most linked hub gene in the CD-specific subnet, and it has an important role in the pathogenesis of Alzheimer’s disease (AD). This relation suggests that some similar genetic factors may be effective in both AD and CD. Last, in order to understand the biological meaning of these disease-specific subnets, they were functionally enriched. The UC-specific modules obtained by the two different approaches reveal common functional properties such as CXC chemokine receptor 1/2 and CXC chemokine, cellular response to transforming growth factor beta (TGF-beta) stimulus, T-cell proliferation involved in immune response, and identical protein binding. The CD-specific modules obtained by the two different approaches reveal common functional properties such as cytokine production, interleukin-1 (IL-1) receptor activity, NF-kappa B (NF-κB) signaling pathway, bladder cancer, and growth factor activity. It is important to note that chemokines—special types of cytokines—and antibacterial response are important in UC-specific subnets, whereas cytokines and antimicrobial responses as well as cancer-related pathways are important in CD-specific subnets. Overall, these findings reveal the differences between the IBD subtypes at the molecular level and can facilitate diagnosis for UC and CD as well as provide potential molecular signatures that are specific to disease subtypes.

## Data Availability Statement

The original contributions presented in the study are included in the article/supplementary material, further inquiries can be directed to the corresponding author/s.

## Author Contributions

SEA conceived the work. SEA and SFM collected, analyzed and interpreted the data, and wrote the manuscript. Both authors contributed to the article and approved the submitted version.

## Conflict of Interest

The authors declare that the research was conducted in the absence of any commercial or financial relationships that could be construed as a potential conflict of interest.

## Publisher’s Note

All claims expressed in this article are solely those of the authors and do not necessarily represent those of their affiliated organizations, or those of the publisher, the editors and the reviewers. Any product that may be evaluated in this article, or claim that may be made by its manufacturer, is not guaranteed or endorsed by the publisher.

## Footnotes

1
http://www.ncbi.nlm.nih.gov/geo

2
http://www.ebi.ac.uk/Tools/webservices/psicquic/view/main.xhtml

3
https://apps.cytoscape.org/apps/keypathwayminer

4
https://apps.cytoscape.org/apps/stringapp

5
https://biit.cs.ut.ee/gprofiler

## References

[B1] Alcaraz N. Kucuk H. Weile J. Wipat A. Baumbach J. (2011). KeyPathwayMiner: detecting case-specific biological pathways using expression data. *Internet Math.* 7 299–313.

[B2] Alcaraz N. List M. Dissing-Hansen M. Rehmsmeier M. Tan Q. Mollenhauer J. (2016). Robust de novo pathway enrichment with KeyPathwayMiner 5. *F1000Res.* 28:1531. 10.12688/f1000research.9054.1 27540470 PMC4965696

[B3] Alcaraz N. Pauling J. Batra R. Barbosa E. Junge A. Christensen A. (2014). KeyPathwayMiner 4.0: condition-specific pathway analysis by combining multiple omics studies and networks with Cytoscape. *BMC Syst. Biol.* 8:99. 10.1186/s12918-014-0099-x 25134827 PMC4236746

[B4] Anka Idrissi D. Senhaji N. Aouiss A. Khalki L. Tijani Y. Zaid N. (2021). IL-1 and CD40/CD40L platelet complex: elements of induction of Crohn’s disease and new therapeutic targets. *Arch. Pharm. Res.* 44 117–132. 10.1007/s12272-020-01296-1 33394309

[B5] Batra R. Alcaraz N. Gitzhofer K. Pauling J. Ditzel H. J. Hellmuth M. (2017). On the performance of de novo pathway enrichment. *npj Syst. Biol. Appl.* 3:6. 10.1038/s41540-017-0007-2 28649433 PMC5445589

[B6] Bruno M. E. C. Rogier E. W. Arsenescu R. I. Flomenhoft D. R. Kurkjian C. J. Ellis G. I. (2015). Correlation of biomarker expression in colonic mucosa with disease phenotype in Crohn’s disease and ulcerative colitis. *Dig. Dis. Sci.* 60 2976–2984. 10.1007/s10620-015-3700-2 25956706 PMC4575253

[B7] Buanne P. Di Carlo E. Caputi L. Brandolini L. Mosca M. Cattani F. (2007). Crucial pathophysiological role of CXCR2 in experimental ulcerative colitis in mice. *J. Leukoc. Biol.* 82 1239–1246. 10.1189/jlb.0207118 17656654

[B8] Burgueño J. F. Fritsch J. González E. E. Landau K. S. Santander A. M. Fernández I. (2020). Epithelial TLR4 signaling activates DUOX2 to induce microbiota-driven tumorigenesis. *Gastroenterology* 160 797.e–808.e. 10.1053/j.gastro.2020.10.031 33127391 PMC7879481

[B9] Burke J. P. Mulsow J. J. O’Keane C. Docherty N. G. Watson R. W. O’Connell P. R. (2007). Fibrogenesis in Crohn’s disease. *Am. J. Gastroenterol.* 102 439–448. 17156147 10.1111/j.1572-0241.2006.01010.x

[B10] Buttó L. F. Schaubeck M. Haller D. (2015). Mechanisms of microbe–host interaction in Crohn’s disease: dysbiosis vs. Pathobiont selection. *Front. Immunol.* 6:555. 10.3389/fimmu.2015.00555 26635787 PMC4652232

[B11] Chen C. Shen H. Zhang L. G. Liu J. Cao X. G. Yao A. L. (2016). Construction and analysis of protein-protein interaction networks based on proteomics data of prostate cancer. *Int. J. Mol. Med.* 37 1576–1586. 10.3892/ijmm.2016.2577 27121963 PMC4866967

[B12] Choi J.-k Kim D.-W. Shin S.-Y. Park E.-C. Kang J.-G. (2016). Effect of ulcerative colitis on incidence of colorectal cancer: results from the nationwide population-based cohort study (2003-2013). *J. Cancer* 7 681–686. 10.7150/jca.14264 27076849 PMC4829554

[B13] Cline M. S. Smoot M. Cerami E. Kuchinsky A. Landys N. Workman C. (2007). Integration of biological networks and gene expression data using cytoscape. *Nat. Protoc.* 2 2366–2382. 10.1038/nprot.2007.324 17947979 PMC3685583

[B14] Cohen L. J. Cho J. H. Gevers D. Chu H. (2019). Genetic factors and the intestinal microbiome guide development of microbe- based therapies for inflammatory bowel diseases. *Gastroenterology* 156 2174–2189. 10.1053/j.gastro.2019.03.017 30880022 PMC6568267

[B15] Dinarello C. A. (2011). Interleukin-1 in the pathogenesis and treatment of inflammatory diseases. *Blood* 117 3720–3732. 10.1182/blood-2010-07-273417 21304099 PMC3083294

[B16] Dinarello C. A. (2019). The IL-1 family of cytokines and receptors in rheumatic diseases. *Nat. Rev. Rheumatol.* 15 612–632. 10.1038/s41584-019-0277-8 31515542

[B17] Du L. Kim J. J. Shen J. Chen B. Dai N. (2017). KRAS and TP53 mutations in inflammatory bowel disease-associated colorectal cancer: a meta-analysis. *Oncotarget* 8 22175–22186. 10.18632/oncotarget.14549 28077799 PMC5400656

[B18] Duerr R. H. Taylor K. D. Brant S. R. Rioux J. D. Silverberg M. S. Daly M. J. (2006). A genome-wide association study identifies IL23R as an inflammatory bowel disease gene. *Science* 314 1461–1463. 10.1126/science.1135245 17068223 PMC4410764

[B19] Ellis R. D. Goodlad J. R. Limb G. A. Powell J. J. Thompson R. P. H. Punchard N. A. (1998). Activation of nuclear factor kappa B in Crohn’s disease. *Inflamm. Res.* 47 440–445. 10.1007/s000110050358 9865503

[B20] Fakhoury M. Al-Salami H. Negrulj R. Mooranian A. (2014). Inflammatory bowel disease: clinical aspects and treatments. *J. Inflamm. Res.* 7 113–120. 10.2147/jir.s65979 25075198 PMC4106026

[B21] Fisher R. C. Bellamkonda K. Alex Molina L. Xiang S. Liska D. Sarvestani S. K. (2019). Disrupting inflammation-associated CXCL8-CXCR1 signaling inhibits tumorigenicity initiated by sporadic- and colitis-colon cancer stem cells. *Neoplasia* 21 269–281. 10.1016/j.neo.2018.12.007 30738331 PMC6370871

[B22] Franceschini A. Szklarczyk D. Frankild S. Kuhn M. Simonovic M. Roth A. (2012). STRING v9.1: protein-protein interaction networks, with increased coverage and integration. *Nucleic Acids Res.* 41 D808–D815. 10.1093/nar/gks1094 23203871 PMC3531103

[B23] Garlanda C. Dinarello C. A. Mantovani A. (2013). The interleukin-1 family: back to the future. *Immunity* 39 1003–1018. 10.1016/j.immuni.2013.11.010 24332029 PMC3933951

[B24] Gathungu G. Zhang C. K. Zhang W. Cho J. H. (2012). A two-marker haplotype in the IRF5 gene is associated with inflammatory bowel disease in a North American cohort. *Genes Immun.* 13 351–355. 10.1038/gene.2011.90 22257839 PMC3809990

[B25] Geng Z. Geng Q. (2021). Risk of urinary bladder cancer in patients with inflammatory bowel diseases: a meta-analysis. *Front. Surg.* 8:636791. 10.3389/fsurg.2021.636791 34124132 PMC8188732

[B26] Griffith J. W. Sokol C. L. Luster A. D. (2014). Chemokines and chemokine receptors: positioning cells for host defense and immunity. *Annu. Rev. Immunol.* 32 659–702. 10.1146/annurev-immunol-032713-120145 24655300

[B27] Han Y. M. Koh J. Kim J. W. Lee C. Koh S.-J. Kim B. (2017). NF-kappa B activation correlates with disease phenotype in Crohn’s disease. *PLoS One* 12:e0182071. 10.1371/journal.pone.0182071 28753650 PMC5533307

[B28] He H. Lin D. Zhang J. Wang Y.-p Deng H.-w (2017). Comparison of statistical methods for subnetwork detection in the integration of gene expression and protein interaction network. *BMC Bioinformatics* 18:149. 10.1186/s12859-017-1567-2 28253853 PMC5335754

[B29] Hudspath C. B. Ellis J. Eum P. Manibusan P. (2018). Tumor protein 63-related disorders and its association with Crohn’s disease. *Am. J. Gastroenterol.* 113:S1179.

[B30] Hugot J. P. Chamaillard M. Zouali H. Lesage S. Cézard J. P. Belaiche J. (2001). Association of NOD2 leucine-rich repeat variants with susceptibility to Crohn’s disease. *Nature* 411 599–603. 10.1038/35079107 11385576

[B31] Jairath V. Feagan B. G. (2019). Global burden of inflammatory bowel disease. *Lancet Gastroenterol. Hepatol.* 5 2–3. 10.1016/s2468-1253(19)30358-9 31648974

[B32] Kaur S. (2013). Brenner’s encyclopedia of genetics | |. *Genomics* 3 310–312. 10.1016/b978-0-12-374984-0.00642-2

[B33] Kim W. Y. (2004). Role of VHL gene mutation in human cancer. *J. Clin. Oncol.* 22 4991–5004. 10.1200/jco.2004.05.061 15611513

[B34] Kobayashi K. Tomita H. Shimizu M. Tanaka T. Suzui N. Miyazaki T. (2017). p53 expression as a diagnostic biomarker in ulcerative colitis-associated cancer. *Int. J. Mol. Sci.* 18:1284. 10.3390/ijms18061284 28621756 PMC5486106

[B35] Koch Hansen L. Sevelsted-Møller L. Rabjerg M. Larsen D. Hansen T. P. Klinge L. (2014). Expression of T-cell KV1.3 potassium channel correlates with pro-inflammatory cytokines and disease activity in ulcerative colitis. *J. Crohns Colitis* 8 1378–1391. 10.1016/j.crohns.2014.04.003 24793818 PMC4216648

[B36] Kuno R. Ito G. Kawamoto A. Hiraguri Y. Sugihara H. Y. Takeoka S. (2021). Notch and TNF-α signaling promote cytoplasmic accumulation of OLFM4 in intestinal epithelium cells and exhibit a cell protective role in the inflamed mucosa of IBD patients. *Biochem. Biophys. Rep.* 25:100906. 10.1016/j.bbrep.2020.100906 33490652 PMC7808948

[B37] Li C. Kuemmerle J. F. (2014). Mechanisms that mediate the development of fibrosis in patients with Crohn’s disease. *Inflamm. Bowel Dis.* 20 1250–1258. 10.1097/MIB.0000000000000043 24831560 PMC4057349

[B38] Li H. Li Q. Sun S. Lei P. Cai X. Shen G. (2020). Integrated bioinformatics analysis identifies ELAVL1 and APP as candidate crucial genes for Crohn’s disease. *J. Immunol. Res.* 2020:3067273. 10.1155/2020/3067273 32724827 PMC7382743

[B39] Lu X. Yu Y. Tan S. (2017). p53 expression in patients with ulcerative colitis–associated with dysplasia and carcinoma: a systematic meta-analysis. *BMC Gastroenterol.* 17:111. 10.1186/s12876-017-0665-y 29070013 PMC5655860

[B40] Lu Y. Li X. Liu S. Zhang Y. Zhang D. (2018). Toll-like receptors and inflammatory bowel disease. *Front. Immunol.* 9:72. 10.3389/fimmu.2018.00072 29441063 PMC5797585

[B41] Magalhaes J. G. Sorbara M. T. Girardin S. E. Philpott D. J. (2011). What is new with Nods? *Curr. Opin. Immunol.* 23 29–34. 10.1016/j.coi.2010.12.003 21190821

[B42] Mahida Y. R. Ceska M. Effenberger F. Kurlak L. Lindley I. Hawkey C. J. (1992). Enhanced synthesis of neutrophil-activating peptide-I/interleukin-8 in active ulcerative colitis. *Clin. Sci.* 82 273–275. 10.1042/cs0820273 1312411

[B43] Meyers T. J. Weiner A. B. Graff R. E. Desai A. S. Cooley L. F. Catalona W. J. (2020). Association between inflammatory bowel disease and prostate cancer: a large-scale, prospective, population-based study. *Int. J. Cancer* 147 2735–2742. 10.1002/ijc.33048 32399975 PMC7577830

[B44] Mitsialis V. Wall S. Liu P. Ordovas-Montanes J. Parmet T. Vukovic M. (2020). Single-cell analyses of colon and blood reveal distinct immune cell signatures of ulcerative colitis and Crohn’s disease. *Gastroenterology* 159 591.e–608.e. 10.1053/j.gastro.2020.04.074 32428507 PMC8166295

[B45] Muthas D. Reznichenko A. Balendran C. A. Böttcher G. Clausen I. G. Kärrman Mårdh C. (2016). Neutrophils in ulcerative colitis: a review of selected biomarkers and their potential therapeutic implications. *Scand. J. Gastroenterol.* 52 125–135. 10.1080/00365521.2016.1235224 27610713

[B46] Nasser M. W. Raghuwanshi S. K. Grant D. J. Jala V. R. Rajarathnam K. Richardson R. M. (2009). Differential activation and regulation of CXCR1 and CXCR2 by CXCL8 monomer and dimer. *J. Immunol.* 183 3425–3432. 10.4049/jimmunol.0900305 19667085 PMC2860795

[B47] Neha P. David H. Nathan M. Sara A. Qihong H. Jack T. R. (2008). MicroRNAs can regulate human APP levels. *Mol. Neurodegener.* 3:10. 10.1186/1750-1326-3-10 18684319 PMC2529281

[B48] Neurath M. F. (2014). Cytokines in inflammatory bowel disease. *Nat. Rev. Immunol.* 14 329–342. 10.1038/nri3661 24751956

[B49] Nissim-Eliraz E. Nir E. Marsiano N. Yagel S. Shpigel N. Y. (2021). NF-kappa-B activation unveils the presence of inflammatory hotspots in human gut xenografts. *PLoS One* 16:e0243010. 10.1371/journal.pone.0243010 33939711 PMC8092666

[B50] Pugliese D. Armuzzi A. Castri F. Benvenuto R. Mangoni A. Guidi L. (2020). TRPM7 is overexpressed in human IBD-related and sporadic colorectal cancer and correlates with tumor grade. *Dig. Liver Dis.* 52 1188–1194. 10.1016/j.dld.2020.05.027 32505565

[B51] Rakshit H. Rathi N. Roy D. (2014). Construction and analysis of the protein-protein interaction networks based on gene expression profiles of Parkinson’s disease. *PLoS One* 9:e103047. 10.1371/journal.pone.0103047 25170921 PMC4149362

[B52] Ran J. Li H. Fu J. Liu L. Xing Y. Li X. (2013). Construction and analysis of the protein-protein interaction network related to essential hypertension. *BMC Syst. Biol.* 7:32. 10.1186/1752-0509-7-32 23587307 PMC3641020

[B53] Rao V. S. Srinivas K. Sujini G. N. Kumar G. N. (2014). Protein-protein interaction detection: methods and analysis. *Int. J. Proteomics* 2014:147648. 10.1155/2014/147648 24693427 PMC3947875

[B54] Raudvere U. Kolberg L. Kuzmin I. Arak T. Adler P. Peterson H. (2019). g:Profiler: a web server for functional enrichment analysis and conversions of gene lists (2019 update). *Nucleic Acids Res.* 47 W191–W198. 10.1093/nar/gkz369 31066453 PMC6602461

[B55] Reimand J. Arak T. Adler P. Kolberg L. Reisberg S. Peterson H. (2016). g:Profiler—a web server for functional interpretation of gene lists. *Nucleic Acids Res.* 44 83–89. 10.1093/nar/gkw199 27098042 PMC4987867

[B56] Renner M. Bergmann G. Krebs I. End C. Lyer S. Hilberg F. (2007). DMBT1 confers mucosal protection in vivo and a deletion variant is associated with Crohn’s disease. *Gastroenterology* 133 1499–1509. 10.1053/j.gastro.2007.08.007 17983803

[B57] Sarrabayrouse G. Landolfi S. Pozuelo M. Willamil J. Varela E. Clark A. (2020). Mucosal microbial load in Crohn’s disease: a potential predictor of response to faecal microbiota transplantation. *EBioMedicine* 51:102611. 10.1016/j.ebiom.2019.102611 31901867 PMC6948165

[B58] Shannon P. (2003). Cytoscape: a software environment for integrated models of biomolecular interaction networks. *Genome Res.* 13 2498–2504. 10.1101/gr.1239303 14597658 PMC403769

[B59] Shih D. Q. Targan S. R. (2007). Immunopathogenesis of inflammatory bowel disease. *World J. Gastroenterol.* 14:390. 10.3748/wjg.14.390 18200661 PMC2679127

[B60] Stallmach A. Giese T. Schmidt C. Ludwig B. Mueller-Molaian I. Meuer S. C. (2004). Cytokine/chemokine transcript profiles reflect mucosal inflammation in Crohn’s disease. *Int. J. Colorectal Dis.* 19 308–315. 10.1007/s00384-003-0554-4 14605835

[B61] Szklarczyk D. Gable A. L. Nastou K. C. Lyon D. Kirsch R. Pyysalo S. (2020). The STRING database in 2021: customizable protein–protein networks, and functional characterization of user-uploaded gene/measurement sets. *Nucleic Acids Res.* 49 D605–D612. 10.1093/nar/gkaa1074 33237311 PMC7779004

[B62] Tatiya-Aphiradee N. Chatuphonprasert W. Jarukamjorn K. (2018). Immune response and inflammatory pathway of ulcerative colitis. *J. Basic Clin. Physiol. Pharmacol.* 30 1–10. 10.1515/jbcpp-2018-0036 30063466

[B63] van Haaften W. T. Blokzijl T. Hofker H. S. Olinga P. Dijkstra G. Bank R. A. (2020). Intestinal stenosis in Crohn’s disease shows a generalized upregulation of genes involved in collagen metabolism and recognition that could serve as novel anti-fibrotic drug targets. *Ther. Adv. Gastroenterol.* 13:175628482095257. 10.1177/1756284820952578 32922514 PMC7457685

[B64] Vennou K. E. Piovani D. Kontou P. I. Bonovas S. Bagos P. G. (2019). Multiple outcome meta-analysis of gene-expression data in inflammatory bowel disease. *Genomics* 112 1761–1767. 10.1016/j.ygeno.2019.09.019 31634529

[B65] Wang S. Wang J. Ma R. Yang S. Fan T. Cao J. (2020). IL-10 enhances T cell survival and is associated with faster relapse in patients with inactive ulcerative colitis. *Mol. Immunol.* 121 92–98. 10.1016/j.molimm.2020.03.001 32193038

[B66] Wang Z. Xu B. Zhang H. Fan R. Zhou J. Zhong J. (2014). Association between STAT3 gene polymorphisms and Crohn’s disease susceptibility: a case–control study in a Chinese Han population. *Diagn. Pathol.* 9:104. 10.1186/1746-1596-9-104 24885273 PMC4047544

[B67] Warby S. C. Visscher H. Collins J. A. Doty C. N. Carter C. Butland S. L. (2011). HTT haplotypes contribute to differences in Huntington disease prevalence between Europe and East Asia. *Eur. J. Hum. Genet.* 19 561–566. 10.1038/ejhg.2010.229 21248742 PMC3083615

[B68] Warsow G. Struckmann S. Kerkhoff C. Reimer T. Engel N. Fuellen G. (2013). Differential network analysis applied to preoperative breast cancer chemotherapy response. *PloS One* 8:e81784. 10.1371/journal.pone.0081784 24349128 PMC3857210

[B69] Watanabe M. Watanabe N. Iwao Y. Ogata H. Kanai T. Ueno Y. (1997). The serum factor from patients with ulcerative colitis that induces T cell proliferation in the mouse thymus is interleukin-7. *J. Clin. Immunol.* 17 282–292. 10.1023/a:1027322631036 9258767

[B70] Wawrzyniak M. Scharl M. (2018). Genetics and epigenetics of inflammatory bowel disease. *Swiss. Med. Wkly.* 148:e00015. 10.4414/smw.2018.14671 30378641

[B71] Williams E. J. Haque S. Banks C. Johnson P. Sarsfield P. Sheron N. (2000). Distribution of the interleukin-8 receptors, CXCR1 and CXCR2, in inflamed gut tissue. *J. Pathol.* 192 533–539. 10.1002/1096-989620009999:9999<::aid-path732<3.0.co;2-x 11113872

[B72] Winter M. W. Weinstock J. V. (2020). “Inflammatory bowel disease,” in *The Autoimmune Diseases*, 6th Edn, Chap. 46, eds Rose N. R. Mackay I. R. (Cambridge: Academic Press), 871–894. 10.1016/b978-0-12-812102-3.00046-4

[B73] Xu X. Fukui H. Ran Y. Wang X. Inoue Y. Ebisudani N. (2019). The link between type III reg and STAT3-associated cytokines in inflamed colonic tissues. *Mediators Inflamm.* 2019:7859460. 10.1155/2019/7859460 31780871 PMC6875322

[B74] Yashiro M. (2014). Ulcerative colitis-associated colorectal cancer. *World J. Gastroenterol.* 20 16389–16397. 10.3748/wjg.v20.i44.16389 25469007 PMC4248182

[B75] Yuan F. Zhang Y.-H. Kong X.-Y. Cai Y.-D. (2017). Identification of candidate genes related to inflammatory bowel disease using minimum redundancy maximum relevance, incremental feature selection, and the shortest-path approach. *BioMed. Res. Int.* 2017:5741948. 10.1155/2017/5741948 28293637 PMC5331171

[B76] Zhang C. Liu S. Peng L. Wu J. Zeng X. Lu Y. (2021). Does inflammatory bowel disease increase the risk of lower urinary tract tumors: a meta-analysis. *Transl. Androl. Urol.* 10 164–173. 10.21037/tau-20-1020 33532306 PMC7844520

[B77] Zhang S. L. Wang S. N. Miao C. Y. (2017). Influence of microbiota on intestinal immune system in ulcerative colitis and its intervention. *Front. Immunol.* 8:1674. 10.3389/fimmu.2017.01674 29234327 PMC5712343

[B78] Zhu Y. Jiang H. Chen Z. Lu B. Li J. Shen X. (2019). Genetic association between IL23R rs11209026 and rs10889677 polymorphisms and risk of Crohn’s disease and ulcerative colitis: evidence from 41 studies. *Inflamm. Res.* 69 87–103. 10.1007/s00011-019-01296-y 31728561

[B79] Zhu Y. Yang S. Zhao N. Liu C. Zhang F. Guo Y. (2021). CXCL8 chemokine in ulcerative colitis. *Biomed. Pharmacother.* 138:111427. 10.1016/j.biopha.2021.111427 33706134

